# Pyogenic liver abscess in pediatric populations in Beijing (2008–2023)

**DOI:** 10.1186/s12879-024-09634-0

**Published:** 2024-07-29

**Authors:** Yue Xie, Ling-yun Guo, Bing Liu, Hui-li Hu, Bing Hu, Tian-ming Chen, Su-yun Qian, Ming-yan Hei, Gang Liu

**Affiliations:** 1 Key Laboratory of Major Diseases in Children, Ministry of Education, Department of Infectious Diseases, National Center for Children’s Health, Beijing Children’s Hospital, Capital Medical University, Beijing, China; 2 Pediatric Intensive Care Unit, National Center for Children’s Health, Beijing Children’s Hospital, Capital Medical University, Beijing, China; 3 Neonatal Center, National Center for Children’s Health, Beijing Children’s Hospital, Capital Medical University, Beijing, China

**Keywords:** Pyogenic liver abscess, Pathogen, mNGS, Antibiotics, Children

## Abstract

**Background:**

Data on pyogenic liver abscess (PLA) of children in China have been limited. We aimed to summarize the clinical feather, microbiological characteristics, management, and outcome of PLA in children.

**Method:**

We retrospectively reviewed PLA cases from January 2008 to June 2023 at Beijing Children’s Hospital. Clinical characteristics, pathogens and management were analyzed.

**Results:**

We diagnosed 57 PLA patients in our center. The median onset age was 4.5 years and the male-to-female ratio was 1.6:1. The median diagnostic time was nine days and the median length of stay was 22 days. Twenty-eight patients (49.1%) had predisposing factors, around 71.4% of the patients had malignant hematology and primary immunodeficiency disease. Patients with underlying factors were more likely to have extrahepatic organ involvement (*p* = 0.024), anemia (*p *< 0.001), single abscess (*p* = 0.042), unilateral involvement (*p* = 0.039), and small size of the abscess (*p* = 0.008). Twenty-four patients (42.1%) had extrahepatic organ involvement. Pathogens were identified in 17 patients (29.8%), the most common pathogens were *Klebsiella pneumoniae* and *Staphylococcus aureus.* The positive rate of metagenomic next-generation sequencing (mNGS) was 87.5% (7/8). On multivariable analysis, the extrahepatic organ involved (*p* = 0.029) and hepatomegaly (*p* = 0.025) were two independent factors associated with poor outcomes.

**Conclusions:**

PLA is usually seen in children with predisposing factors. Malignant hematology and primary immunodeficiency disease were the most common underlying diseases. Extrahepatic organ involvement and hepatomegaly are associated with poor prognosis. Increased use of mNGS could be beneficial for identifying pathogens.

**Supplementary Information:**

The online version contains supplementary material available at 10.1186/s12879-024-09634-0.

## Introduction

Pyogenic liver abscess (PLA) is considered an uncommon infection that comprises the majority of hepatic abscess (HA) [1] According to the limited reports on children, the incidence of PLA is higher in developing countries. For instance, the morbidity was 78.9 per 100,000 admissions in South India [2]. Nevertheless, the incidence of PLA is reported to be lower among children in developed countries, with 25 per 100,000 admissions in the US [3], and 2.9 per 100,000 in Sweden [4]. And in Taiwan, was 8.9 to 20 per 100,000 admissions [5, 6].

Overall, *Staphylococcus aureus* (*S. aureus*) is frequently isolated from PLA in children and accounts for nearly 20–50% of cases. *Escherichia coli (E. coli), Klebsiella pneumoniae (K. pneumoniae),* and *Enterobacter* are also common pathogens [7–9]. In recent years, investigators found the prevalence of *K. pneumoniae* in PLA increased worldwide. Similarly, an upward tendency was shown in China [8]. Patients with inducing factors tend to get PLA. Diabetic mellitus (DM), cirrhosis, and immunocompromised state are well-known predisposing factors [10].

But so far, most studies focused on adults but seldom children, which resulted in limited data in our country. Here, we present a single-center study of PLA and analyze clinical characteristics, pathogens and management in the pediatric population.

## Patients and methods

### Study population

In this retrospective study, we reviewed data from children (younger than 18 years old) who were admitted to Beijing Children’s Hospital (National Center for Children’s Health, China; A 970-bed tertiary pediatric hospital) and diagnosed PLA from January 2008 to June 2023. We collected information on the risk factors, clinical presentations, and laboratory tests including pathogen results, radiological findings, management, and outcomes of PLA patients.

### Inclusion criteria

All of the patients met at least one of the following inclusion criteria: (1) Presence of abscess in the liver on imaging examinations and reduced after anti-bacterial treatment; (2) Bacterial culture positive from aspiration of liver abscess.

The definition of extrahepatic involvement: Except for the liver, infections were found in other tissues or organs through CT, MRI, ultrasound, or cerebrospinal fluid examination.

### Statistical analysis

Categorical variables were presented as numbers and percentages, while continuous variables were shown as the median and interquartile range (IQR). Categorical variables were compared using the Chi-square or Fisher’s exact tests as appropriate. Continuous variables within two groups were compared using the Mann–Whitney U test according to their distribution. We used univariable logistic regression to evaluate the risk factors of the poor outcome, when considering factors with *p*-value < 0.05, multivariable logistic regression was made. The odds ratio (OR) and confidence interval at 95% (CI95%) were presented. *P*-value < 0.05 was considered significant. All of the statistical analyses were conducted using Statistical Product and Service Solutions (SPSS), version 23.0 (IBM, NY, USA).

## Results

Fifty-seven children were diagnosed as PLA from January 2008 to June 2023, including 35 males (61.4%), the median age of onset was 4.5 (0.7, 8.4) years, and four cases were neonates. The median diagnostic time was 9 (4, 15) days, and the median length of stay was 22 (14, 31) days (Table 1).

**Table 1:** Clinical characteristics of children with PLA

Characteristics	Value
Male:female	35:22
Age (years), median	4.5(0.7–8.4)
Time to diagnosis (day), median	9(4–15)
Hospital stay (day), median	22(14–31)
Predisposing factor, *n* (%)	28 (49.1)
Extrahepatic infection lesion, *n* (%)	24 (42.1)
Multiple extrahepatic infection lesions, *n* (%)	14 (24.6)
Symptoms and signs, *n* (%)	
Fever	51 (89.5)
Hepatomegaly	18 (31.6)
Abdominal pain	14 (24.6)
Blood tests, median	
WBC (10,000/μL)	13.6 (11.7–17.4)
PCT (ng/mL)	0.8 (0.2–3.2)
CRP (mg/L)	85 (40–120)
ALT (U/L)	22 (13–56)
Imaging findings	
Solitary, *n* (%)	24 (42.1)
Unilateral lobar involved, *n* (%)	38 (66.7)
Right lobe, *n* (%)	31 (54.4)
Size (cm), median	2.5 (1.0–5.6)
Defined pathogen, *n* (%)	17 (29.8)
Ultrasound-guild aspiration, *n* (%)	16 (28.1)
Favorable outcome, *n* (%)	50 (87.7)
ICU admission, *n* (%)	14 (24.6)

*WBC* white blood cell, *PCT* procalcitonin, *CRP* C-reactive protein, *ESR* erythrocyte sedimentation rate, *ALT* transaminase, *ICU* intensive care unit

### Clinical presentations

**Fig. 1:**
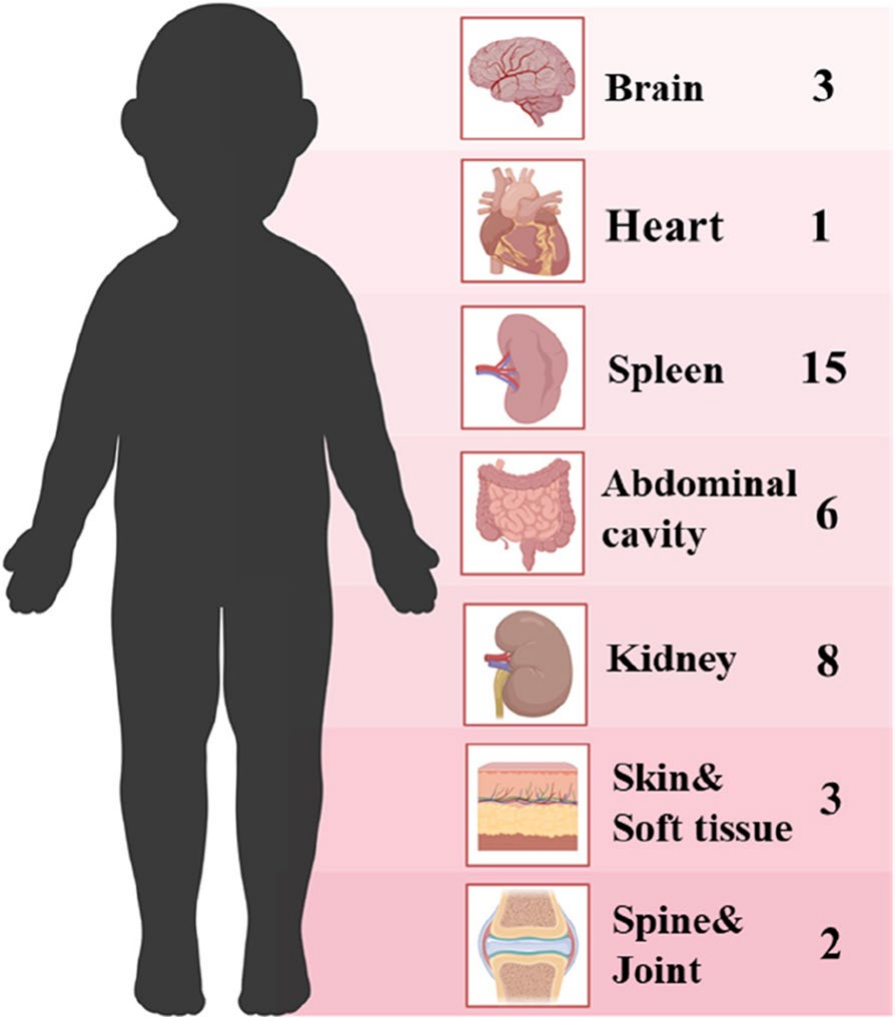
Distribution of extrahepatic organ involved

Fifty patients (87.7%) initially presented with fever, 13 patients (22.8%) had abdominal pain, four patients (7.0%) experienced cough and four patients (7.0%) had diarrhea. Extrahepatic involvement was observed in 24 patients (42.1%). The splenic abscess was the most common with 15 patients (26.3%), eight patients (14.0%) had kidney abscess, six patients (10.5%) had intra-abdominal infections, and other complications such as meningitis, skin infection, endocarditis, infection of hip joint, soft tissue and spine were also observed (Fig. 1). Significantly, 14 patients (24.6%) had more than two infection foci apart from liver abscess.

Among four neonates, case 1 had fever after umbilical vein catheterization, case 2 was asymptomatic with elevated CRP after umbilical vein catheterization, case 3 had fever with abdominal distension, and case 4 only had abdominal distension.

### Predisposing factors

Twenty-eight patients (49.1%) had underlying factors, over two-thirds (67.9%, *n* = 19) were in an immunosuppressed state. Eleven patients (19.3%) received a long-time use of immunosuppressive agents, including nine cases of hematological malignancy (five acute lymphoblastic leukemia, two lymphoma, one hemophagocytic syndrome and one chronic active Epstein-Barr virus infection), the rest were one case of systemic lupus erythematosus and inflammatory bowel disease separately. Eight patients (14.0%) had primary immunodeficiency disease (PID), including five combined immunodeficiency, congenital neutropenia, hyper IgE syndrome and chronic granulomatous disease (CGD) one case each. Appendicitis was found in four patients (7.0%), two of them had perforations. Two neonates were associated with a history of undergoing umbilical vein catheter insertion. Type 1 DM, hypertyrosinemia and glycogen storage disease were observed in the remaining three cases separately.

Patients with predisposing factors were more likely to have extrahepatic involved (*p* = 0.024), anemia (*p* < 0.001), single abscess (*p* = 0.042), unilateral involvement (*p* = 0.039), and small size of the abscess (*p* = 0.008)(Table 2).

**Table 2:** Comparison of clinical characteristic between the patients with or without predisposing factor

	With predisposing factor (*n* = 28)	Without predisposing factor (*n* = 29)	*P*
Male, n (%)	21 (75.0)	14 (48.3)	0.038
Age (years), median	3.8 (0.6–7.0)	6.0 (1.2–9.0)	0.508
Time to diagnosis (day), median	9 (4–13)	7 (5–18)	0.754
Hospital stay (day), median	24 (16–27)	22 (13–37)	0.866
Extrahepatic infection lesion, *n* (%)	16 (57.1)	8 (27.6)	0.024
Multiple extrahepatic infection lesions, *n* (%)	6 (21.4)	8 (27.6)	0.589
Symptoms and signs			
Fever, *n* (%)	22 (78.6)	28 (96.6)	0.096
The highest temperature, median	39.1 (39–39.6)	39.5 (39–40)	0.301
Hepatomegaly, *n* (%)	10 (35.7)	9 (31.0)	0.708
Imaging findings			
Single abscess, *n* (%)	8 (28.6)	16 (55.2)	0.042
Unilateral lobar involved, *n* (%)	15 (53.6)	23 (79.3)	0.039
Size(cm), median	1.8 (0.6–3.5)	4.2 (1.9–6.4)	0.008
Blood tests			
WBC (10,000/μL)	12.7 (7.6–17.9)	13.7 (12.6–15.7)	0.278
HGB (g/L)	92 (81–108)	116 (107–127)	< 0.001
PCT (ng/mL)	0.8 (0.2–6.1)	0.47 (0.05–2.79)	0.201
CRP (mg/L)	77.3 (42.1–121.5)	89 (35.5–120)	0.842
ALT (U/L)	19.2 (12.5–94.6)	29.8 (16.4–46.8)	0.377
ICU admission, *n* (%)	10 (35.7)	4 (13.8)	0.055
Poor outcome, *n* (%)	6 (21.4)	1 (3.4)	0.096

*WBC* white blood cell, *HGB* hemoglobin, *PCT* procalcitonin, *CRP* C-reactive protein, *ALT* transaminase, *ICU *intensive care unit

## Laboratory data

### Blood tests

C-reactive protein (CRP) level was elevated (> 8 mg/L) in 54 patients (94.7%), with a median of 85 (40, 120) mg/L. Increased procalcitonin (PCT) level (> 0.05 ng/mL) was observed in 34 patients (87.2%, *n* = 39), with a median of 0.8 (0.2, 3.2) ng/mL. Leukocytosis (white blood cell > 10,000/μL) was seen in 46 patients (80.7%), with a median of 13,600 (11,700,17,400) /μL. Seventeen patients (29.8%) had elevated level of alanine transaminase (> 40U/L), with a median of 22 (13, 56) U/L.

### Imaging findings

All patients performed ultrasound, 55 patients (96.5%) presented with low hypoechoic lesions in ultrasound, and two patients were normal. Forty-two patients (73.7%) performed the contrast-enhanced computed tomography (CECT) or magnetic resonance imaging (MRI) scan. Lesions of low density with edge intensified showed on CECT, hypointensity on T1 and T2 weighted image with edge intensified showed on contrast-enhanced MRI. Thirty-three patients (57.9%) had multiple abscesses. Thirty-eight patients (66.7%) had unilateral involvement and most patients (81.6%, *n* = 31) occupied the right lobe. The median abscess diameter was 2.5 (1.0, 5.6) cm.

### Pathogen findings

In the present study, pathogens were identified in 17 patients (29.8%), and two patients had co-infections (Tables 3 and 4). The positive rate of blood culture was 14.5% (8/55). Sixteen patients (28.1%) finished ultrasound-guild aspiration and pus culture, the positive rate was 25.0% (4/16). Seven (12.3%) abscess samples and one blood sample were sent for mNGS, the positive rate was 87.5% (7/8), mNGS increased the diagnostic rate of pathogen by 10.5% (6/57).

**Table 3:** Pathogens defined from enrolled cases. The overall pathogen detection and different method to identify pathgens

Bacteria	N	Blood culture	Pus culture	mNGS
Gram-positive cocci	7	4	1	3
*S.aureus*^*a*^	4	3	1	1
*Streptococcus agalactiae*	1	1		
*Streptococcus intermedius*	1			1
*Bartonella henselae*	1			1
Gram-negative bacillil	12	5	3	5
*K. pneumoniae*^*b*^	5	3	3	
*P. aeruginosa*	3	1		2
*E. coli*	2	1		1
*Fusobacterium nucleatum*	1			1
*Serratia marcescens*	1			1
Total	17^c^	8^d^	4	7^e^

*S. aureus: Staphylococcus aureus, E. coli: Escherichia coli, K. pneumoniae: Klebsiella pneumoniae, P. aeruginosa:Pseudomonas aeruginosa*

^a^One had growth from blood culture and defined by mNGS of pus.

^b^One had growth from pus culture and blood culture.

^c^Two patients were mixed infection. One was *K. pneumoniae* with *S.aureus;* one was *E. coli* with *P. aeruginosa.*

^d^One was defined *K. pneumoniae* with *S.aureus* by blood culture.

^e^One was defined *P. aeruginosa* and *E. coli* by mNGS of blood.

**Table 4:** The distribution of pathogens in patients with underlying disease and without underlying disease

Bacteria	n	With predisposing factor (*n* = 28)	Without predisposing factor (*n* = 29)
Gram-positive cocci	7	2	5
*S.aureus*	4	2	2
*Streptococcus agalactiae*	1		1
*Streptococcus intermedius*	1		1
*Bartonella henselae*	1		1
Gram-negative bacillil	12	8	4
*K. pneumonia*	5	3	2
*P. aeruginosa*	3	3	
*E. coli*	2	2	
*Fusobacterium nucleatum*	1		1
*Serratia marcescens*	1		1
Total	17^*a*^	8^*a*^	9

^a^Two patients had co-infection. One was *K. pneumoniae* with *S.aureus;* one was *E. coli* with *P. aeruginosa*

Four patients were *K. pneumoniae* (one patient was positive for extended-spectrum beta-lactamase and carbapenemases). Three patients were *S.aureus* (one was methicillin-resistant and others were methicillin-susceptible)*.* Two patients had mixed infections. One was *K. pneumoniae* (negative of extended-spectrum beta-lactamase and carbapenemases) with *S.aureus* (methicillin-susceptible) isolated from blood culture, another one was *Escherichia coli* with *Pseudomonas aeruginosa* identified by mNGS*.*

Notably, before 2018, only 18.2% (4/22) of patients performed aspiration and the positive rate of pathogen detection was 9.1% (2/22). After that, with the increased use of mNGS and ultrasound-guided aspiration (34.3%, 12/35), the positive rate of pathogen detection increased to 42.9% (15/35).

### Therapy and outcomes

All patients received intravenous antimicrobial therapy (Tables 5 and 6). Twenty-nine patients (50.9%) were prescribed a single agent as initial treatment, and cefoperazone sulbactam/piperacillin/tazobactam (*n* = 14, 24.6%) was the most common recipe followed by meropenem (*n* = 10, 17.5%). Meropenem combined with vancomycin or linezolid (*n* = 14, 24.6%) was the most common combination therapy regimen. Sixteen patients (28.1%) changed antimicrobial treatment, and more than half (*n* = 9, 56.3%) were correlated with positive pathogen results.

**Table 5:** Antibiotic treatment and outcome of children with PLA. The overall antibiotic use and outcome

Initial anti-infection treatment	*N* (%)	Changed antibiotics (*n*, %)			Died (*n*, %)
		Total	Due to pathogen defined	Due to poor prognosis	
SCF based	22 (38.6)	4 (18.2)	3 (13.6)	1 (4.5)	2 (9.1)
SCF /TZP	14 (24.6)	2 (14.3)	2 (14.3)	0 (0)	0 (0)
SCF + VA /LZD/MTZ	8 (14.0)	2 (25.0)	1(12.5)	1(12.5)	2 (25.0)
MEM based	24 (42.1)	7 (29.2)	4 (16.7)	3 (12.5)	5 (20.8)
MEM	10 (17.5)	3 (30.0)	1 (10.0)	2 (20.0)	1 (10.0)
MEM + VA /LZD	14 (24.6)	4 (28.6)	3 (21.4)	1 (7.1)	4 (28.6)
CRO based	8 (14.0)	4 (50.0)	2 (25.0)	2 (25.0)	0 (0)
CRO	2 (3.5)	2 (100.0)	1 (50.0)	1 (50.0)	0 (0)
CRO + MTZ	4 (7.0)	1 (25.0)	0 (0)	1 (25.0)	0 (0)
CRO + VA /LZD	2 (3.5)	1 (50.0)	1 (50.0)	0 (0)	0 (0)
Others*	3 (5.3)	1 (33.3)	0 (0)	1 (33.3)	0 (0)
Total	57 (100.0)	16 (28.1)	9 (15.8)	7 (12.3)	7 (12.3)

*SCF* cefoperazone/sulbactam, *TZP* piperacillin/tazobactam, *VA* vancomycin, *LZD* linezolid, *MTZ* metronidazole, *MEM* meropenem, *CRO* ceftriaxone, *RFP* rifampicin, *AZM* azithromycin

^*^ VA = 2; cefamandole = 1

**Table 6:** Antibiotic changed due to pathogen defined

No	Pathogen	Initial anti-infection treatment	Changed antibiotics
1	*Streptococcus intermedius*	SCF	SCF + LZD
2	*Serratia marcescens*	SCF	MEM
3	*Fusobacterium nucleatum*	SCF + VA + MTZ	SCF + MTZ
4	*K. pneumoniae*	MEM + VA	SCF
5	*K. pneumoniae*	MEM + VA	SCF
6	*P. aeruginosa* + *E. coli*	MEM + VA	SCF
7	*MRSA*	MEM	MEM + VA
8	*Bartonella henselae*	CRO	RFP + AZM
9	*Streptococcus agalactiae*	CRO + LZD	LZD

*K. pneumoniae: Klebsiella pneumoniae; P. aeruginosa: Pseudomonas aeruginosa; E. coli: Escherichia coli;*

*MRSA* methicillin-resistant *staphylococcus aureus*

Fourteen (24.6%) patients had ever been treated in the intensive care unit. Finally, 50 patients (87.7%) took a favorable turn. Patients in the death group were more likely to have extrahepatic organ involved and hepatomegaly than the survival group (Table 7). On multivariable analysis, the extrahepatic organ involved [OR 13.134 (1.301–32.438), *p* = 0.029] and hepatomegaly [OR 8.893 (1.312–13.296), *p* = 0.025] were two independent factors that lead to poor outcomes.

**Table 7:** Comparison of clinical characteristic between the patients in survival and death group

	Survival (*n* = 50)	Death (*n* = 7)	*P*
Time to diagnosis (day), median	7 (4–13)	12 (9–25)	0.131
Extrahepatic infection lesion, *n* (%)	18 (36)	6 (85.7)	0.037
Multiple extrahepatic infection lesions, *n* (%)	11 (22)	3 (42.9)	0.464
Predisposing factors	22 (44)	6 (85.7)	0.096
Symptoms and signs			
The highest temperature, median	39.3 (39–39.8)	39.4 (39–40)	0.479
Hepatomegaly, *n* (%)	13 (26)	5 (71.4)	0.047
Imaging findings			
Single abscess, *n* (%)	23 (46)	1 (14.3)	0.237
Unilateral lobar involved, *n* (%)	36 (72)	2 (28.6)	0.064
Size(cm), median	2.8 (1.1–5.9)	1.2 (0.7–3.1)	0.156
Blood tests			
WBC (10,000/μL)	13.5 (11.6–17.8)	13.6 (11.8–17.1)	0.874
HGB (g/L)	109 (93.5–122)	88 (76–104)	0.053
PCT (ng/mL)	0.68 (0.11–2.30)	2.8 (0.2–22.3)	0.069
CRP (mg/L)	84 (37–119)	91.2 (74–160)	0.215
ALT (U/L)	23.7 (15–47.3)	13.5(9.2–126.7)	0.246

*WBC* white blood cell, *HGB* hemoglobin, *PCT* procalcitonin, *CRP* C-reactive protein *ALT* transaminase

## Discussion

The age at diagnosis was reported 13 years in the USA [11], 10 years in the UK [12], and 9.6 years in Taiwan [13]. Excluding the data on newborns in our study, the median age of pediatricians with PLA was 5.0 years and 79.2% of the total were under 10 years old, which was younger compared with other studies. Possibly it was related to a high portion of patients with hematological malignancy and primary immunodeficiency disease, which had an early onset age.

Predisposing factors play a vital role in the development of PLA and it differs between adults and children. Immunocompromised state (such as PID, malignancy, use of an immunosuppressive agent and chemotherapy), appendicitis with or without perforation, hepatobiliary disease (including congenital abnormalities, liver cirrhosis, liver transplant), DM, trauma and umbilical venous catheterization were showed to be closely related with PLA in several studies [10, 11, 13, 14]. In adults, the main predisposing factors were diabetes, malignancy and hepatobiliary diseases [15, 16]. However, PLA often occurs in children with PID, especially for selective IgA deficiency, T-cell defect, and CGD [17–19]. In a nationwide population-based analysis, it was almost three times increased risk among children with immunodeficiency [11]. Unlike other factors compared with adults, PID is almost unique to children which can be recognized via personal and family history, detection of immune function, and gene sequencing. Although only one case had a history of DM in our study, this factor should not be ignored. DM has been regarded as a potential risk factor for PLA with a hazard risk of 3.6 to ninefold and co-existence rates of 23–36.6% [20–22]. The mechanism was identified to be neutrophil chemotaxis and phagocytosis impaired, which leads to the weakening of the immune system.

PLA in neonates is rare, and the clinical manifestations and predisposing factors were often distinct from older children. Usually present with fever, abdominal distension and even asymptomatic. Diagnosis in newborns relies more on blood tests, imaging examinations and reaction of anti-infective therapy. In our study, two cases did not have LA on admission, developed fever and raised CRP level after umbilical venous catheterization and finally diagnosed PLA. It is worth noting that ascending infection through umbilical venous catheterization can be a potential cause of PLA in a neonatal period [23]. When a neonate suffers progressive sepsis, PLA should be considered, especially with the history of this operation. In addition, omphalitis could be a very rare cause of PLA in neonates [24].

Clinical manifestations of PLA are nonspecific. Fever and abdominal pain are the most common clinical features and basis for the diagnosis of PLA [12]. Four patients in our study had a cough accompanied with pneumonia. Previous studies reported several patients had a cough because of the stimulation of the diaphragm through a liver abscess nearby, which can even be the only symptom besides fever [25]. Six patients were diagnosed by routine test without any symptoms, one patient was neonate and five had hematological malignancy, half of them had normal CRP level and white blood cell counts. Patients are more susceptible to abscess formation due to long-term immunosuppressive therapy and secondary neutropenia, which could be explained as sometimes asymptomatic at onset. Meanwhile, only three cases with hematological malignancy did not show increased CRP. Which are easy to misdiagnose during clinical assessment and could be partially explained the diagnostic time seemed long in our study.

In the past few decades, *S.aureus* has been the leading casual pathogen isolated from PLA in children all over the world [8, 12]. In recent years, *K. pneumoniae* has become the main pathogen of PLA whether in adults or children, and is closely related to DM, particularly in Asia [13, 26, 27]. Chung et al. from Korea revealed that 78.2% of PLA patients were caused by *K. pneumoniae* [28]. A study conducted by Yeh et al. reported that *K. pneumoniae* was accused of a 36.4% positive blood culture rate and a 64.3% positive pus culture rate in pediatrics [13]. In a meta-analysis of pathogen distribution with PLA in China, Klebsiella spp had an upward tendency during an 11-year period and the highest pooled proportion even reached 71%, meanwhile 66% of patients with DM was related to PLA [8]. One case in our study who suffered from DM was isolated *K. pneumoniae* from pus culture, therefore *K. pneumoniae* should be given priority when PLA patients had DM. In addition, in our study we found PLA patients with underlying disease (mainly in hematological malignancy) were more susceptible to gram negative bacterial infection and co-infections, especially for *Pseudomonas aeruginosa* and *E. coli.*

Our study showed a relatively low positive rate of blood culture (14.5%) and pus culture (25.0%). Culture-based techniques are the golden standard, the low positive rate of culture in our study could be attributed to sample collection after prior antibiotic treatment of pre-hospitalization. It is worth noting that the broad use of ultrasound-guild aspiration and mNGS leads to increase in the positive rate in the detection of pathogens. Seven patients were confirmed pathogens by mNGS. Significantly, there have been no previous reports of identifying *Serratia marcescens, Fusobacterium nucleatum,* and *Bartonella henselae* in PLA patients through mNGS. They were rare pathogens of PLA because of difficulty to identify through conventional methods. Furthermore, co-infection was identified by mNGS in one case. A review indicated that mNGS was proved valuable for the detection of rare and complex pathogens in culture-negative and undiagnosed cases [29]. A study in our center about the evaluation of mNGS for the pathogenic diagnosis showed the ability to identify pathogens from abscess and less affected by prior antibiotics [30, 31]. Zhang et al. reported that compared with the conventional method (57.5%) and culture (45.2%), mNGS (86.3%) showed a better evaluation of diagnostic ability in focal infection [31]. In addition, mNGS could better achieve a rational use of antibiotics (antimicrobial de-escalation and completely antibiotic regimen change). Applying this technology in clinical practice to gain more experience is our direction in the future.

According to our study, 50% of patients had an anti-infection regimen changed based on ceftriaxone and the least change presented when patients received cefoperazone/sulbactam or piperacillin/tazobactam based treatment. Currently, PLA is absent of empirical antibiotic guidelines. Empiric antibiotics should cover *Streptococcus, staphylococcus*, *K. pneumoniae*, *E.coli* and anaerobes, which are commonly seen in pediatric PLA [11].

In our study, almost all dead patients had predisposing factors and extrahepatic organs involved. Four patients died of multiple organ failure caused by serious infection and three patients died of progression of underlying diseases, suggesting that early diagnosis and treatment of disseminated infections and control of underlying diseases were extremely important to improve prognosis. The mortality rate of PLA in pediatrics has decreased from nearly 15% in the 1980s to even null, which was considerably lower than in adults [3, 11, 27]. Research indicated about 15.7% of HA patients develop complications, which can be accused of most death [32]. A study showed that age-related leukocytosis, neutrophilia, elevated aspartate transaminase or alanine transaminase, hypoalbuminemia and abscess size of more than 80 cc at presentation are predictors of poor outcomes in pediatric liver abscess [33].

In conclusion, PLA is more likely to occur in patients with predisposing factors, especially in an immunosuppressed state. Patients with extrahepatic organ involvement and hepatomegaly were relative with poor prognosis. The combined complication of mNGS and culture could increase the positive rate of pathogen detection.

### Supplementary Information

Supplementary Material 1.

## Data Availability

The datasets analyzed during the current study are available from the corresponding author upon reasonable request.

## Abbreviations

PLA: Pyogenic liver abscess
HA: Hepatic abscess
mNGS: Metagenomic next-generation sequencing
*S. aureus*: *Staphylococcus aureus*
*E. coli*: *Escherichia col* *i*
*K. pneumonia*: *Klebsiella pneumonia*
DM: Diabetic mellitus
IQR: Interquartile range
OR: Odds ratio
CI95%: Confidence interval at 95%
WBC: White blood cell
PCT: Procalcitonin
CRP: C-reactive protein
ESR: Erythrocyte sedimentation rate
ALT: Transaminase
ICU: Intensive care unit
PID: Primary immunodeficiency disease
CGD: Chronic granulomatous disease
HGB: Hemoglobin
*P. aeruginosa*: *Pseudomonas aeruginosa*
CECT: Contrast-enhanced computed tomography
MRI: Magnetic resonance imaging
SCF: Cefoperazone/sulbactam
TZP: Piperacillin/tazobactam
VA: Vancomycin
LZD: Linezolid
MTZ: Metronidazole
MEM: Meropenem
CRO: Ceftriaxone
RFP: Rifampicin
AZM: Azithromycin
MRSA: Methicillin-resistant *s**taphylococcus aureus*
