# Supplementary material for: Pyogenic liver abscess in pediatric populations in Beijing (2008–2023)
Source: BMC Infect Dis. 2024 Jul 29;24:745. doi: 10.1186/s12879-024-09634-0 (PMC11285452; doi:10.1186/s12879-024-09634-0)
Supplement: Supplementary file 1 — Supplementary Material 1. [file 12879_2024_9634_MOESM1_ESM.docx]

The method of metagenomic next-generation sequencing for diagnosis pathogen

DNA extraction
DNA was extracted directly from the 300ul blood or pus sample (each patient and negative “no-template” control) using the TIANamp Micro DNA Kit (DP316, Tiangen Biotech, Beijing, China). The sample was added proteinase K (10ml) and 300 ml buffer GB (with carrier RNA) and then incubated at 56 °C (10 min). After 300 ul cold absolute ethyl alcohol was added and the tube was incubated at room temperature (5 min). Transferring the liquid to a new adsorption column and the liquid was washed with buffer GD and buffer PW. The DNA was dissolved in 40ul of Tris–ethylenediaminetetraacetic acid buffer.
Library generation, and sequencing
The extracted DNA was sonicated with a Bioruptor Pico device to generate 200–300 bp fragments. According to the standard protocol of the BGISEQ-500 sequencing platform (BGI-Tianjin, Tianjin, China), DNA libraries were constructed through end repaired, adapter added overnight and polymerase chain reaction amplification to the extracted DNA. Quality control was carried out using a bioanalyser (Agilent 2100, Agilent Technologies, Santa Clara, CA, USA) combined with quantitative PCR to measure the adapters before sequencing. DNA sequencing was then performed using the BGISEQ-500 platform (BGI-Tianjin, Tianjin, China).

Data processing and analysis
High quality sequencing data was generated after removal of short (< 35 bp) reads, low quality and low complexity reads. The readings were then mapped to the human reference genome (hg19 and YH sequences) using the Burrows-Wheeler Aligner. The remaining data were aligned with the NCBI microbial genome database (ftp://ftp.ncbi.nlm.nih.gov/genomes/), which included the genome sequences of 3446 bacterial species (104 species of Mycobacterium tuberculosis and 45 species of mycoplasma/chlamydia), 1515 viral species, 206 fungal species and 140 parasites connected to human diseases. The mapped data were used for further analysis. The depth and coverage of each species was calculated using Soap Coverage on the SOAP website (http://soap.genomics.org.cn/).
